# Supplementary material for: Turning preference in dogs: North attracts while south repels
Source: PLoS One. 2021 Jan 28;16(1):e0245940. doi: 10.1371/journal.pone.0245940 (PMC7842976; doi:10.1371/journal.pone.0245940)
Supplement: S1 Table — ITP = index of turning preference; W-N designates the combination in which the test dishes were placed west and north; N-E = designates the combination in which the test dishes were placed north and east; E-S designates the combination in which the test dishes were placed east and south; S-W = designates the combination in which the test dishes were placed south and west, mN = 0° designates a control experiment; mN = 90° designates an experimental condition with a shifted magnet field. (DOCX) [file pone.0245940.s001.docx]

**S1 Table**

|  | **mN= 0°** | | | | | **mN= 90°** | | | | |
| --- | --- | --- | --- | --- | --- | --- | --- | --- | --- | --- |
| **Dog** | **ITP**  **W-N** | **ITP**  **N-E** | **ITP**  **E-S** | **ITP**  **S-W** | **ITP**  **all** | **ITP**  **W-N** | **ITP**  **N-E** | **ITP**  **E-S** | **ITP**  **S-W** | **ITP**  **all** |
| Amalka | 33.33 | 16.67 | 0.00 | 16.67 | 16.67 | 33.33 | 66.67 | 100.00 | 100.00 | 75.00 |
| Arthur | -20.00 | 40.00 | -70.00 | -80.00 | -32.50 | -10.00 | -60.00 | -90.00 | -70.00 | -57.50 |
| Azizi | -33.33 | -33.33 | 50.00 | 0.00 | -4.17 | 83.33 | 0.00 | 66.67 | 16.67 | 41.67 |
| Barca | 66.67 | -16.67 | 0.00 | 16.67 | 16.67 | 83.33 | 33.33 | 83.33 | 66.67 | 66.67 |
| Bertik | -16.67 | -66.67 | -100.00 | -83.33 | -66.67 | -33.33 | 0.00 | -100.00 | 16.67 | -29.17 |
| Bessy | 100.00 | 50.00 | 40.00 | 60.00 | 62.50 | -10.00 | 50.00 | -10.00 | 60.00 | 22.50 |
| Figy | 33.33 | 0.00 | -50.00 | 0.00 | -4.17 | 33.33 | -33.33 | 66.67 | 16.67 | 20.83 |
| Gofi | -100.00 | -90.00 | -100.00 | -100.00 | -97.50 | -100.00 | -100.00 | -90.00 | -90.00 | -95.00 |
| Hard | -10.00 | -20.00 | -30.00 | -10.00 | -17.50 | 0.00 | 40.00 | 60.00 | -10.00 | 22.50 |
| Hugo | 16.67 | 16.67 | -50.00 | -16.67 | -8.33 | 16.67 | -33.33 | -66.67 | -16.67 | -25.00 |
| Hurvinek | -16.67 | -50.00 | -66.67 | -50.00 | -45.83 | -50.00 | -50.00 | -50.00 | -33.33 | -45.83 |
| Jimmy | 90.48 | 70.00 | 10.00 | 70.00 | 60.49 | -20.00 | 0.00 | 0.00 | 60.00 | 10.00 |
| Kacka | 0.00 | 0.00 | -66.67 | 0.00 | -16.67 | 16.67 | -66.67 | 23.08 | -50.00 | -9.68 |
| Kuky | 33.33 | 16.67 | 66.67 | 83.33 | 50.00 | 66.67 | 16.67 | -16.67 | 50.00 | 29.17 |
| Naty | 10.00 | -50.00 | -90.00 | -50.00 | -45.00 | -40.00 | -70.00 | -100.00 | -90.00 | -75.00 |
| Offi | 33.33 | 0.00 | -33.33 | 50.00 | 15.38 | 16.67 | -16.67 | -33.33 | 33.33 | 0.00 |
| Pecka | 16.67 | 0.00 | 0.00 | 50.00 | 16.67 | 16.67 | 16.67 | 33.33 | 100.00 | 41.67 |
| Plysak | 16.67 | -83.33 | -100.00 | 0.00 | -41.67 | -83.33 | -50.00 | -100.00 | -83.33 | -79.17 |
| Punta | -50.00 | -33.33 | -84.62 | -50.00 | -55.10 | -16.67 | 0.00 | 0.00 | -33.33 | -12.50 |
| Roxxy | 50.00 | 83.33 | 66.67 | 50.00 | 62.50 | 83.33 | 66.67 | 66.67 | 66.67 | 70.83 |
| Shedy | 60.00 | 50.00 | -20.00 | 20.00 | 27.50 | 80.00 | 20.00 | 60.00 | 60.00 | 55.00 |
| Sisi | 33.33 | 0.00 | 33.33 | -16.67 | 12.50 | 66.67 | 83.33 | 33.33 | 100.00 | 70.83 |
| Zofka | 66.67 | 16.67 | 50.00 | 83.33 | 54.17 | 50.00 | -16.67 | 50.00 | 66.67 | 37.50 |
