# Supplementary material for: Turning preference in dogs: North attracts while south repels
Source: PLoS One. 2021 Jan 28;16(1):e0245940. doi: 10.1371/journal.pone.0245940 (PMC7842976; doi:10.1371/journal.pone.0245940)
Supplement: S2 Table — (DOCX) [file pone.0245940.s002.docx]

**S2 Table**

| **Countable variables** | **Characteristic** | |
| --- | --- | --- |
| Age of the dog (years, mean, SE) | 4.83 | 0.59 |
| Order of the test series 1 to 5 (min, max) | 1 | 5 |
| **Categorical variables** | | |
| Name of dog | 23 levels | |
| Breed | 5 levels | |
| Sex of the dog | Male or Female | |
| Magnetic coil | magnetic coil switched on and magnetic coil switched off | |
| Turning preference classes | Right-preferring, Left-preferring, and Irresolute | |
