# Supplementary material for: Turning preference in dogs: North attracts while south repels
Source: PLoS One. 2021 Jan 28;16(1):e0245940. doi: 10.1371/journal.pone.0245940 (PMC7842976; doi:10.1371/journal.pone.0245940)
Supplement: Attachment — Submitted filename: Adamkova response to reviewers.pdf [file pone.0245940.s005.pdf]

Dear Prof. Roman,

we are pleased about the positive views of the reviewers and appreciate also the careful editing of the manuscript by the reviewer #1:

We accepted all but one suggestions:

Lines 153-162 — paragraph should be in past tense
Corrected

Line 219 tense shift
Corrected

Line 244 “according to based on” (redundant)
We have not changed this sentence, because "based on first trials" is here a complementary information, a fact, which should be pointed out

Line 326 “more frequently" need to add "chosen”
Corrected

In the figure caption for Fig 1 please give a sense of what these numbers mean. - ie - left and + right? We don't need the full explanation that comes later in the text, but a sense of what these numbers are telling us would be helpful at this point.
The reviewer means probably Table 1 and/or Figure 3. We complemented the caption in both cases as suggested.

I still find the figures confusing, but I’m not an expert on how to present these results with turning preference so I leave it to the other reviewer and the editor's expertise.
We assure the reviewer that this way of illustrating the results is common in the literature dealing with spatial orientation.

Once again, many thanks for considering our manuscript and ist academic processing.

With best regards and wishes for a prosperous and healthy new year

Hynek Burda

Hynek Burda
On behalf of all coauthors.
